# Harnessing AI and Quantum Computing for Revolutionizing Drug Discovery and Approval Processes: Case Example for Collagen Toxicity

**DOI:** 10.2196/69800

**Published:** 2025-07-22

**Authors:** David Melvin Braga, Bharat Rawal

**Affiliations:** 1 Department of Quantum Computing, Capitol Technology University, Laurel, MD, United States; 2 Department of Quantum Computing, Grambling State University, 403 Main Street, Grambling, LA, 71245, United States, 1 318-274-2421, 1 318-274-2421

**Keywords:** generative AI, quantum computing, computational data, new drug discovery, computer-aided drug discovery, artificial intelligence

## Abstract

Artificial intelligence (AI) and quantum computing will change the course of new drug discovery and approval. By generating computational data, predicting the efficacy of pharmaceuticals, and assessing their safety, AI and quantum computing can accelerate and optimize the process of identifying potential drug candidates. In this viewpoint, we demonstrate how computational models obtained from digital computers, AI, and quantum computing can reduce the number of laboratory and animal experiments; thus, computer-aided drug development can help to provide safe and effective combinations while minimizing the costs and time in drug development. To support this argument, 83 academic publications were reviewed, pharmaceutical manufacturers were interviewed, and AI was used to run computational data for determining the toxicity of collagen as a case example. The research evidence to date has mainly focused on the ability to create computational in silico data for comparison to actual laboratory data and the use of these data to discover or approve newly discovered drugs. In this context, “in silico” describes scientific studies performed using computer algorithms, simulations, or digital models to analyze biological, chemical, or physical processes without the need for laboratory (in vitro) or live (in vivo) experiments. Digital computers, AI, and quantum computing offer unique capabilities to tackle complex problems in drug discovery, which is a critical challenge in pharmaceutical research. Regulatory agents will need to adapt to these new technologies. Regulatory processes may become more streamlined, using adaptive clinical trials, accelerating pathways, and better integrating digital data to reduce the time and cost of bringing new drugs to market. Computational data methods could be used to reduce the cost and time involved in experimental drug discovery, allowing researchers to simulate biological interactions and screen large compound libraries more efficiently. Creating in silico data for drug discovery involves several stages, each using specific methods such as simulations, synthetic data generation, data augmentation, and tools to generate, collect, and affect human interaction to identify and develop new drugs.

## Introduction

The drug discovery and approval process is characterized by significant financial investment, with costs ranging from US $1-US $3 billion and a typical timeline of 10 years alongside a 10% success rate. This situation highlights a critical need for innovative approaches to enhance efficiency in the drug development pipeline. Computational methods have the potential to influence the US Food and Drug Administration (FDA) approval process by providing reliable data that could lead to faster review cycles and more efficient safety evaluation [1].

Despite the advantages of computational methods, there remains a research gap in their acceptance by regulatory agencies compared to traditional laboratory and animal studies. International Organization for Standardization (ISO) 10993‐5 serves as the standard for assessing the cytotoxicity of materials and the necessity for a robust foundation to validate computational models within a regulatory framework.

Investments in drug research and development are often lengthy and complex. Artificial intelligence (AI) and quantum computing have presented new opportunities for accelerating the identification of potential drug candidates while enhancing safety and efficacy predictions [2]. Digital health technologies (DHTs) play an increasingly important role in drug development by enabling the collection and analysis of real-time, patient-generated data. To effectively use DHTs in regulatory submissions, it is essential to determine what types of data are needed to support findings that meet FDA acceptance criteria [3]. These data may include genomic information, side effect profiles, and timelines associated with drug development, all of which can accelerate and refine the evaluation of new therapeutics [4].

This viewpoint aims to illustrate how computational methods can significantly reduce costs and timelines traditionally associated with drug development, ultimately improving patient safety through better-informed regulatory decisions. Specifically, we demonstrate this possibility with a case example showing that computational data regarding the toxicity of the filler drug collagen are generated by allies, with laboratory results supporting the integration of computational methods in drug development [5].

## Use Cases of Drug Discovery With AI and Quantum Computing

### Role of AI in the Discovery of New Drugs

Investments in new drug development are a long and complex process of drug research and development; however, with the advancement of AI, technology has emerged as a leading tool in analyzing potential new drugs. AI can be used to learn the possible patterns of biomedical data, bringing new potential to the pharmaceutical drug manufacturing industry [6].

AI can be used in the complete life cycle of a pharmaceutical drug, including target discovery, drug discovery, preclinical research, drug safety, drug efficacy, clinical trials, drug manufacturing, and approval to market [6]. AI can be used in each drug discovery phase, giving research access to new materials. New data are constantly being added to the drug repositories. Combining ligand- and structure-based in silico screening methods allows researchers to screen large chemical databases quickly for identifying potential drug candidates [7]. Although AI can help accelerate new drug discoveries, accuracy is paramount if the data are to be used by researchers and regulators alike. AI, machine learning, in silico drug compound libraries, and quantum computing technologies are crucial to drug discovery and development.

### Use of AI for Target Identification of New Drugs

AI systems can analyze diverse data types such as genetic, proteomic, and clinical data to identify potential therapeutic targets. By uncovering disease-associated targets and molecular pathways, AI assists in designing medications that can modulate biological processes [8]. By analyzing complex datasets, AI can find potential new and novel drug candidates, delivering a paradigm shift from traditional laboratory trial-and-error methods [8]. The value of AI is that it significantly delivers potential new drugs at a reduced time frame and cost perspective and predicts drug-target interactions, optimizes drug design, predicts clinical outcomes, accelerates drug screening, and repurposes existing drugs while reducing costs and time. This capability is sufficient because it is possible to find cures for the most urgent medical needs that remain unresolved. Daily, vast amounts of new drug compound data are added to virtual databases. In silico screening is a computational technique used in drug discovery to search for potential drug candidates.

### Virtual Screening of New Drugs

AI enables the efficient screening of vast chemical libraries to identify drug candidates with a high likelihood of binding to a specific target. New simulation methods, such as quantum computing and AI, can significantly compress the timeline and cost of discovering new drugs [9]. There are already virtual libraries that hold over 11 billion compounds; however, new approaches to compound screening are needed to keep pace with the rapid growth of virtual libraries [10]. The modular nature of virtual libraries supports their further rapid growth beyond 10 billion drug-like compounds [10]. By simulating chemical interactions and predicting binding affinities, AI helps researchers prioritize and select compounds for experimental testing, saving time and resources. Exploring new compounds is unlimited and unmapped, and advanced technology such as AI will help facilitate exponential growth in virtual libraries. Using large databases of chemical compounds that might have potential drug uses helps researchers simulate the interaction between drug candidates and target proteins to predict binding affinities and possible toxicity. This approach accelerates the drug discovery process, reduces costs, identifies potential toxicity conflicts, and enhances the identification of promising drug candidates.

### Molecular Docking for New Drugs

For in silico screening to be cost-effective and efficient, compound libraries that include known drug-like molecules must be built. Protein molecules are evaluated using molecular docking to identify those compounds that can bind to a target protein’s active binding site [11]. Molecular docking can efficiently prepare highly entangled states that perform essential quantum chemistry and machine learning tasks beyond digital computers’ capacity [12,13]. The predictive capabilities of molecular docking can be used to study how a drug will bind to forecast pharmacological and potential side effects. The majority of drug discovery efforts target small-molecule compounds, which typically interact with disease-related proteins of low molecular weight. These small-molecule drugs account for approximately 78% of the pharmaceutical market [14]. Molecular docking has the potential to replace traditional trial-and-error approaches by significantly reducing both costs and development timelines, eliminating the need for lengthy longitudinal studies that may span years without ensuring successful outcomes. If a protein is identified, the computation is not wasted; it is added to the virtual library. Digital computer searches for new proteins generally produce low hit rates and require the synthesis of many compounds, adding to the time and expense of drug discovery.

### Molecular Modeling

Traditional computing methods struggled to accurately simulate quantum effects in huge molecules. Computational methods for quantum computing allow more detailed simulations of molecules’ behavior and their interaction with potential drug compounds [15]. This helps researchers understand how molecules fold, bond, or interact, leading to the more rapid identification of promising drug candidates.

Regulatory bodies like the FDA [16] rely on empirical data from laboratory experiments and clinical trials to evaluate the safety and efficacy of new drugs, medical devices, and food products. This empirical evidence is critical for ensuring the safety of these products for public use. Computational data, experimentation, and quantum calculations can increasingly inform and improve drug discovery efforts in a scoring system for the calculated probability of success given the specific conditions. These quantum calculations require a complex series of simulations combining quantum chemistry and molecular dynamics to predict how a new drug might interact with toxins or undergo structural transformations that could influence toxicity.

### ISO 10993 Computational Data for Prebiocompatibility

ISO 10993‐5 is the corresponding test for determining the cytotoxicity of materials. Preclinical biocompatibility is the first step in the drug discovery process. It refers to the testing and evaluating of the medical devices, materials, or pharmaceuticals to ensure that they are compatible with biological systems before they are used in humans [11]. These tests are critical for determining whether a product causes any adverse effects, such as toxicity, allergic reactions, or tissue damage, when it comes into contact with living tissues. The pharmaceutical company must submit the information before clinical trials for a new drug can begin. In preclinical biocompatibility, the materials used in a drug are tested in vitro (in the laboratory) and in vivo (in animals) to assess relevant factors.

## Contribution of the Paper

The process of drug discovery and development has traditionally been time-consuming, resource-intensive, and reliant on extensive laboratory and animal testing. Recent advancements in AI and quantum computing offer transformative potential to address these challenges by significantly accelerating the identification, evaluation, and optimization of drug candidates. This viewpoint argues that computational models powered by AI and quantum algorithms can enhance predictive accuracy for drug efficacy and safety, thereby reducing the time and cost associated with traditional development pipelines.

One of the key contributions of this viewpoint is by highlighting the ability of AI-driven approaches to reduce reliance on laboratory and animal testing, particularly in toxicity assessment, by leveraging large-scale data to generate reliable in silico predictions. Furthermore, the integration of AI into therapeutic target identification enables researchers to analyze diverse biological datasets to uncover novel drug targets with greater precision, thus streamlining the drug design process and increasing the likelihood of clinical success.

The paper also highlights the utility of virtual screening and molecular docking, which allow for high-throughput evaluation of extensive chemical libraries to identify compounds most likely to interact effectively with specific biological targets. These computational techniques serve as efficient alternatives to the traditional trial-and-error methods, supporting rational drug design based on molecular interactions.

Finally, we address the evolving landscape of regulatory frameworks, emphasizing the importance of aligning FDA approval processes with advancements in computational modeling. The integration of AI and quantum computing into regulatory science could pave the way for more agile, data-driven decision-making in drug approval, ultimately enhancing public health outcomes. The main contributions are as follows:

1. Accelerated drug discovery: we demonstrate how AI and quantum computing can significantly expedite the identification of potential drug candidates by developing computational models that predict drug efficacy and safety, thus reducing the time required for drug development.
2. Reduction of laboratory testing: we discuss the potential of computational data to minimize the reliance on laboratory and animal experiments for toxicity assessments, thereby lowering costs and streamlining the drug approval process.
3. Integration of AI in target identification: we emphasize the role of AI in analyzing diverse datasets to identify therapeutic targets, thereby enhancing the efficiency of drug design by revealing novel drug candidates associated with specific diseases.
4. Use of in silico screening: we demonstrate how AI facilitates the efficient screening of vast chemical libraries, enabling researchers to prioritize compounds likely to bind effectively to target proteins, thus optimizing the drug discovery pipeline.
5. Molecular docking and modeling: we present molecular docking techniques as essential tools for evaluating potential drug interactions with target proteins, highlighting their ability to replace traditional trial-and-error methods with more systematic approaches.
6. Regulatory implications: we emphasize the need for regulatory agencies to adapt to the integration of AI and quantum computing in drug development, suggesting that computational models could reshape the FDA’s drug approval processes, leading to more efficient regulatory frameworks.

## Theoretical Framework and Related Work

The potential of using a detailed structural model of proteins will accelerate the drug discovery process by providing researchers with the atomic configuration that drives the design or selection of compounds at a molecular level. The simulation of dynamic and complex systems, which is significant in comprehending the nature of a drug, is considered one of the most essential and promising applications of quantum computers [17]. Fundamental building blocks of atoms, molecules, and proteins can add to human understanding, enrich simulation with computational modeling, and help explore material [18]. Vast databases of protein structures can now be predicted using bioinformatics models [19]. Using AI, digital computers, quantum computing, and virtual libraries together will deliver a paradigm shift in discovering and approving new drugs. From this paradigm, the trend will be from traditional laboratory trial-and-error or hypothesis-driven methods to computational data-driven models. This paradigm will expand the potential for predicting and understanding potential new drugs at a molecular level to understand drug interactions, toxicity, and efficacy.

Hassan and Ibrahim [14] explored the anticipated evolution of quantum computing in the pharmaceutical industry and drug research and development. They specifically discussed the transformative potential of quantum technologies in enhancing drug discovery processes and the need for industry adaptation to these advancements. Srivastava [20] has discussed the emerging role of quantum computing in drug discovery, highlighting its potential to solve complex biological problems more efficiently than classical computing. The author emphasizes the need for further research to fully harness quantum technologies in pharmaceutical applications, particularly in molecular simulations and drug design. Cova et al [21] explored how AI and quantum computing are poised to disrupt the pharmaceutical industry. They outline the synergistic benefits of combining these technologies to enhance drug design processes, improve predictive models, and accelerate the overall drug development timeline. Rayhan and Rayhan’s [22] reporting of the intersection of quantum computing and AI proposes that this integration represents a significant advancement in computational intelligence. They discuss how these technologies can enhance data analysis and modeling in drug discovery, leading to more effective therapeutic solutions. Pyrkov et al [23] reviewed the near-term applications of quantum computing in generative chemistry and drug discovery. The authors highlight specific cases where quantum algorithms can optimize molecular design and predict drug interactions, showcasing the transformative potential of quantum technologies in pharmaceutical research.

Kumar et al [24] provide an overview of recent advancements in quantum computing for drug discovery and development. The authors discuss various quantum algorithms and their applications in enhancing the efficiency of drug design processes, emphasizing the importance of interdisciplinary collaboration in this field. Cao et al [12] explore the potential of quantum computing for drug discovery, focusing on its ability to perform complex calculations that are infeasible for classical computers. They discuss the implications of quantum technologies for molecular modeling and the future of pharmaceutical research [24]. Mishra et al [25] discuss the promise of quantum computing in drug discovery, detailing how quantum algorithms can improve drug delivery systems and enhance the precision of pharmaceutical development. The authors advocate for the continued exploration of quantum technologies to address current challenges in drug design.

Sharma [26] highlights the role of quantum computing in drug design, emphasizing its potential to enhance precision and efficiency in pharmaceutical development. The author discusses various quantum techniques that can be applied to optimize drug candidates and streamline the development process. Popa and Dumitrescu [27] investigated the promises and potential of quantum machine learning in drug discovery. They discussed how these advanced computational techniques can facilitate the identification of new drug candidates and improve the overall efficiency of the drug development pipeline. Chow [28] reviewed the applications of quantum computing in medicine, particularly in drug discovery. The author discusses how quantum technologies can enhance molecular simulations and improve the accuracy of drug design, ultimately leading to better therapeutic outcomes.

## Case Example: Using AI to Determine the Drug Toxicity of Collagen

Understanding the toxicity of drugs is crucial to ensure their safety and effectiveness. Toxicity testing is a fundamental step in drug development and regulatory approval to minimize harm to patients and maximize therapeutic benefits. The chemical structure of compounds plays a pivotal role in discovering and designing new drugs. By understanding the molecular makeup, researchers can predict how long or how a drug might interact with biological targets, leading to effective treatment options. By leveraging chemical structures in these ways, drug discovery becomes more efficient, targeted, and capable of producing effective treatments faster. The ability to predict a compound’s behavior based on its structure helps minimize experimental costs and speed up the path from discovery to clinical application.

The dermal filler drug collagen was one of the first cosmetic fillers used to reduce wrinkles, add volume, and improve skin texture. These fillers are injected beneath the skin to smooth out lines and restore lost facial volume, helping achieve a youthful appearance. Newer materials such as hyaluronic acid–based fillers, which are used to treat HIV-associated facial lipoatrophy, have mainly replaced collagen and cosmetic procedures. However, collagen fillers still offer benefits in specific cases. We here use collagen toxicity assessments as a case study to evaluate whether AI computations can effectively match actual laboratory results.

**Figure 1.**
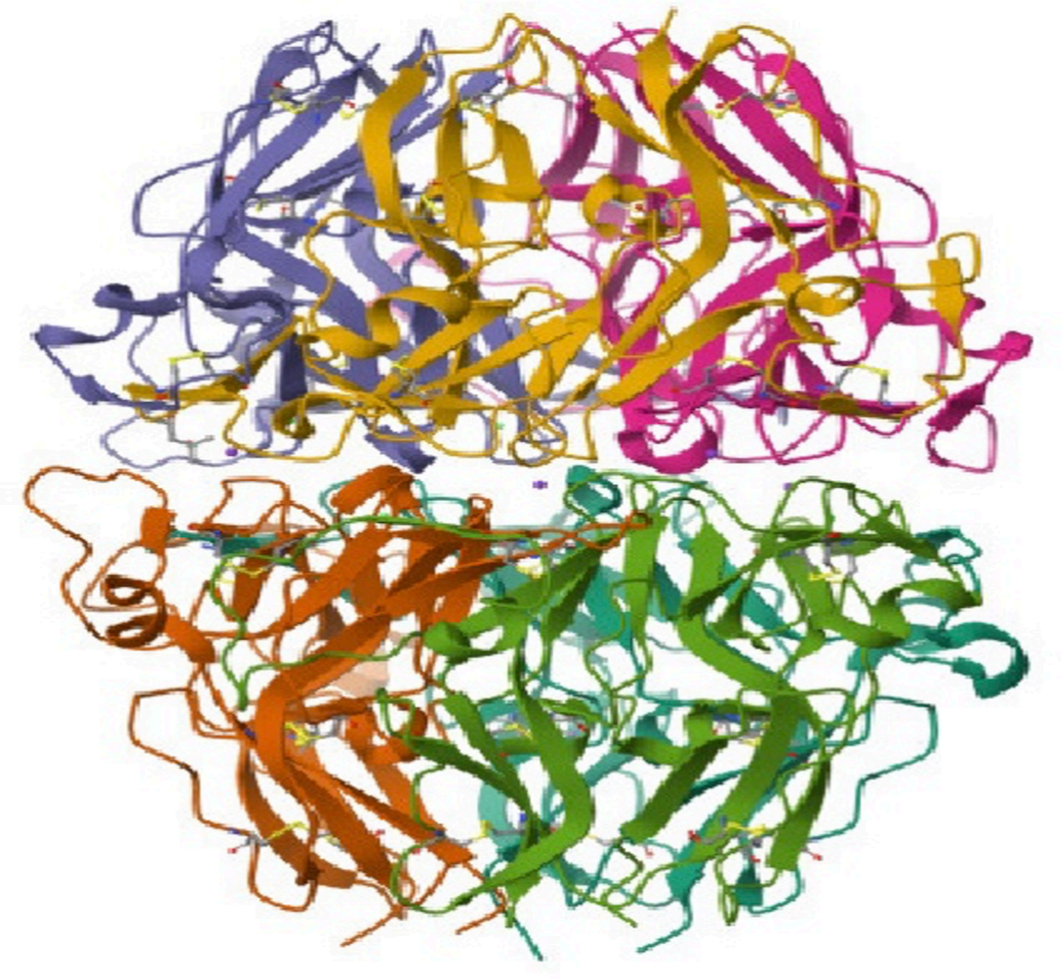
Crystal structure of type IV collagen from bovine.

The chemical structure must be known to compute the toxicity of collagen (Figures1  2).

**Figure 2.**
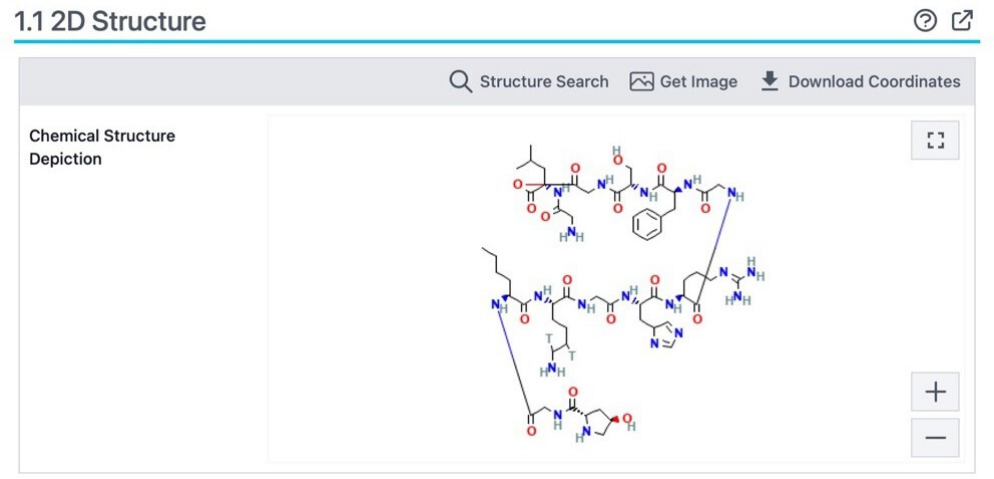
Chemical structure depiction of collagen molecular arrangement and stability.

Collagen is a large and complex protein. Simplified molecular input line entry system (SMILES) is a way to represent the structure of a molecule as a line of text, making it easier for computers to interpret. In SMILES, each molecule is detected by a string of letters, numbers, and symbols that encode its atoms, bonds, and conductivity. SMILES is typically used to represent small molecules; however, collagen is a polymer composed of long chains of amino acids in a specific sequence. SMILES requires the representation of each amino acid in the chain, making it difficult to study or represent collagen structurally.

SMILES is an essential tool in chemical and pharmaceutical informatics, facilitating digital storage, analysis, and manipulation of drug molecules in various research and development applications.

Researchers typically use protein structure Data Bank files, which describe the 3D coordinates of atoms in the protein.

(3H)C(CC(C@@H)(C(=O)NCC(=O)N(C@@H)(CC1C=NC=N1)C(=O)N(C@@H)(CCCN=C(N)N)C(=O)NCC(=O)N(C@@H)(CC2=CC=CC=C2)C(=O)N(C@@H)(CO)C(=O)NCC(=O)OC(=O)(C@H)(CC(C)C)NC(=O)CN)NC(=O)(C@H)(CCCC)NC(=O)CNC(=O)(C@@H)3C(C@H)(CN3)O)C([3H))N

The molecular formula of collagen is *C_57_H_91_N_19_O_16_* [29].

### Using Quantum Computations to Determine the Drug Toxicity of Collagen

Traditional computing methods struggle to simulate quantum effects in molecules, especially huge ones, accurately. Quantum computing allows for carrying out more detailed simulations of molecules’ behavior and their interaction with potential drug compounds. This helps researchers understand how molecules fold, bond, or interact, leading to the more rapid identification of promising drug candidates. Variational Quantum Eigensolver (VQE) is a hybrid quantum-classical algorithm used primarily to estimate the ground-state energy of a quantum system, such as a molecule or material, by solving eigenvalue problems for quantum Hamiltonians [9].

Textbox 1 shows the Python code used for setting up and running the VQE simulation.

Textbox 1 **Python code for Variational Quantum Eigensolver simulation.**

- Define a glycine-proline-hydroxyproline fragment as a molecule.

   For simplicity, we use approximate coordinates for the atoms.

   molecule =Molecule (

   geometry= ([

   ("N", (0.0, 0.0, 0.0)),

   ("C", (1.0, 0.0, 0.0)),

   ("C", (2.0, 1.0, 0.0),

   ("O", (2.0, 2.0, 0.0),),

   ("H", (-0.5, -0.5, 0.5),

   #Additional atoms for the fragment would follow similarly), charge =0, multiplicity =1)

- Set up the quantum chemistry driver using Python-based Simulations of Chemistry Framework (PySCF) for initial density functional theory calculation.
  driver =PySCFDriver (molecule =molecule, basis=“sto3g”) # Use small basis set for simplicity
- Set up the electronic structure problemes_problem =ElectronicStructureProblem(driver)
- Map the problem to qubits using a qubit converter and Jordan-Wigner transformation
  Qubit_converter = QubitConverter[mapper =JordanWignerMapper()
  The optional process is to apply a core orbital freezing transformation to reduce the number of qubits
  transformer =FreezeCoreTransformer() es_problem =transformer.transform(es_problem)
- Set up the ansatz and optimizer for VQE (Variational Quantum Eigensolver)
  # EfficientSU2 is a standard hardware-efficient ansatz with two-qubit entanglement
  ansatz =EfficientSU2(qubit_converter.num_qubis, entanglement=“full”, reps =2)
  optimizer =COBYLA (maxiter =500)
- Define the quantum instance (statevector simulator) to simulate the VQE quantum_instance = QuantumInstance[backend =Aer.get_backend[“sttevector_simulator”]]
- Set up the VQE solver with the ansatz, optimizer, and quantum instance.
  vqe_solver =VQE [ansatz =ansatz, optimizer =optimizer, quantum_instance =quantum_instance] calc =GroundStateEigensolver[qubit_converter, vqe_solver]
- Compute the ground-state energy of the collagen fragment
  result =calc.solve[es_problem]
  Display the computed ground-state energy print[“Computed ground state energy for glycine-proline-hydroxyproline fragment:", result.total_energ

The step-by-step explanation of the code is provided in Textbox 2.

Textbox 2. **Detailed explanation of each step of the Python code.**

- Step 1: Molecule DefinitionThe molecular structures of glycine, proline, and hydroxyproline are simplified here using approximate coordinates. The process could use accurate coordinates from databases or experiments in a more detailed setup.

- Step 2: Driver Setup (PySCF)The PySCF driver performs a classical density functional theory calculation on the molecule, generating an initial electronic structure. Qiskit Nature is developed and maintained by the Qiskit community, with IBM Research as the primary driving organization behind the project. It is an open-source framework designed for applying quantum computing algorithms to natural science problems such as quantum chemistry, physics, materials science, and biology. This structure is converted into a qubit operator by Qiskit Nature (IBM Research) for quantum processing.

- Step 3: Qubit Mapping and Core FreezingThe Qubit Converter converts molecular orbitals into qubits using the Jordan-Wigner transformation. Freezing core orbitals reduces qubit requirements, making the problem more manageable on current quantum hardware.

- Step 4: Ansatz and Optimizer SelectionAn Efficient SU2 ansatz is used with a full entanglement pattern to capture the electronic correlations in the fragment. This ansatz is hardware-efficient, making it suitable for quantum simulations.

- Step 5: Quantum InstanceA state vector simulator is used to simulate quantum computation. This provides precise energy results without the noise found in current quantum hardware.

- Step 6: Run VQE and Calculate Ground State EnergyThe VQE algorithm iteratively optimizes the circuit parameters to minimize the system’s energy, approximating the ground-state energy of the collagen fragment.

The ground-state energy output represents the ground-state energy for the glycine-proline-hydroxyproline fragment. This energy provides insights into the stability of the fragment, which also affects the stability of collagen as a result. The potential extensions and next steps are as follows:

1. Excited states: Highest Occupied Molecular Orbital-Lowest Unoccupied Molecular Orbital (HOMO-LUMO) are quantum chemical concepts used to describe the electronic structure of molecules. Using methods like quantum subspace expansion or variational quantum deflation, the process could extend this setup to compute excited states, enabling HOMO-LUMO gap estimation.
2. Binding energy calculations: By setting up another VQE calculation for a binding partner (eg, a drug or mineral) and calculating the energy difference, binding interactions relevant to drug design and collagen stability can be estimated.
3. Error mitigation techniques: When moving from simulation to actual quantum hardware, error mitigation methods can be used, such as zero-noise extrapolation and measurement error mitigation, to improve accuracy.

Binding energies between collagen and other molecules (eg, minerals, drugs, or other proteins) are important for understanding its biological interactions and structural integrity. Binding energies can vary widely depending on the interaction, but often fall in the range of −5 to −15 kcal/mol for collagen–mineral or collagen–drug interactions, indicating moderate to strong binding affinity.

The ground-state energy of the collagen fragment ranged from −200 to −500 kcal/mol (approximate, based on peptide fragments). The HOMO-LUMO gap was calculated to be 5–8 eV, suggesting stability. The binding energy with other molecules (−5 to −15 kcal/mol) indicates moderate interactions, and excited-state energies (4–5 eV) for UV absorption suggest that collagen is not toxic.

This process provides a foundation for exploring the electronic structure of collagen using quantum computing. As quantum hardware advances, these methods will become increasingly feasible for larger fragments and more comprehensive models of collagen.

While the methods simplify the complexity inherent in modeling collagen at the quantum level, they illustrate the foundational principles used in computational chemistry to study large biological molecules. Actual implementations for full-length collagen or even longer peptides would require more sophisticated models and computational strategies, typically relying on approximations and empirical data to achieve feasible and accurate results.

### Using Laboratory Methods to Determine the Drug Toxicity of Collagen

An increasing number of soft tissue filler substances are introduced to the beauty market outside the United States, which often needs more experimental and clinical data to support their claim. Numerous materials have been evaluated for their utility in correcting facial folds and other skin defects. Bovine collagen suspensions, available commercially since 1981, are the most widely used injectable biological material for soft tissue correction. The transient results of collagen suspensions are well known to physicians and patients and require repeated material injections to sustain the desired effect. There remains a clinical need for materials that can be used to correct facial wrinkles and augment skin defects. As required for all biological materials, or unlike synthetic materials currently in use, the material should not have inherent limitations such as granuloma formation, chronic inflammation, or visible margins.

The collagen used in dermal fillers is typically atelocollagen, which consists of 3 separate helix-shaped α-chains (polypeptide chains) that wrap around each other and form a 3-stranded helix. Amino acid analysis shows that this is collagen type 1. Each polypeptide chain contains about 1000 cross-linked amino acids. The collagen molecule consists of 2 identical polypeptides, α-1(1), and a third polypeptide chain that has a different amino acid sequence, a-2(1). The individual polypeptide chains can be separated by sodium dodecyl sulfate-polyacrylamide gel electrophoresis.

A dermal filler is indicated for correcting contour deficiencies of soft tissue. Wrinkles develop because the thickness of the skin’s dermal layer significantly diminishes during aging. As a case example, we consider a dermal filler composed of absolutely round and smooth polymethyl methacrylate (PMMA), a synthetic polymer widely used in medical, industrial, and cosmetic applications. The filler comprises PMMA microspheres, 30–42 microns in size, suspended in a water-based carrier gel containing 3.5% bovine collagen, 96.5% buffered isotonic water for injection, and 0.3% lidocaine [30].

The PMMA microspheres are suspended in a solution of partly denatured 3.5% bovine collagen. Following injection of the filler, the collagen vehicle is absorbed by the body within 1–3 months, during which the nondegradable PMMA microspheres stimulate the body to encapsulate each sphere with the patient’s collagen. This results in a long-lasting correction of wrinkles and other soft tissue defects [30]. Bovine collagen is converted to atelocollagen by treatment with pepsin to remove the peptide ends, thus reducing its antigenic potential [31].

From the Artes Laboratory report [30], the toxic metal content in the syringe of the semi-permanent dermal filler product Artecoll was determined as follows (Table 1): lead (Pb)=0.03 μg, chromium (Cr)=0.14 μg, cadmium (Cd)=0.017 μg, and mercury (Hg)<0.006 μg per 0.5 g Artecoll. The concentrations of lead, chromium, cadmium, and mercury were reported to be 0.057 ppm, 0.259 ppm, 0.030 ppm, and 0.010 ppm, respectively. This indicates that not only are the individual concentrations of each heavy metal in Artecoll well below 1 ppm, but the combined total of all heavy metals is also less than 0.4 ppm. As a result, the risk of releasing toxic levels of heavy metals from Artecoll is considered negligible [30]. The current permissible exposure limit for chromium was found to be 1 mg/m^3^ TWA. The LD50 (median lethal dose) of chromium trioxide subcutaneously injected into a dog was 330 mg/kg body weight [1]. Approximately 1 g of potassium dichromate is considered a lethal dose preceded by gastrointestinal bleeding and massive fluid loss [5]. The revised Immediately Dangerous to Life or Health (IDLH) level for chromium was set to 250 mg Cr/m^3^ air [30].

**Table 1.** Component specifications for 3.5% atelocollagen.

Parameter	Specification	Method
Collagen (calculated from hydroxyproline)	3.0‐4.0%	Spectrophotometry
Hydroxyproline	0.41‐0.55%	Spectrophotometry
Lidocaine HCI^a^	0.27‐0.33%	HPLC^b^
Heavy metals	<20 ppm	DAB 10^c^
pH	6.8‐7.8	DAB 10
Pyrogenicity	<36.25 EU/ml	DAB 10
Sterility	Sterile	DAB 10

a HCI: hydrochloric acid; a strong, corrosive acid commonly used in chemical reactions, laboratory testing, and pH control.

b HPLC: high-performance liquid chromatography; an analytical technique used to separate, identify, and quantify components in a mixture, widely applied in pharmaceuticals, environmental analysis, and biochemistry.

c DAB-10: 10-deacetyl baccatin.

The results of the toxicological laboratory data show no evidence that acute exposure to a high chromium concentration would cause irreversible health effects within 30 minutes (Table 2) [32].

**Table 2.** Polymethyl methacrylate heavy metals specifications list the daily requirement of chromium.

Item	Specification
Cd	<0.1 ppm
Hg	<0.1 ppm
Pb	<0.2 ppm

The duration of a collagen toxicity test can vary depending on the type and scope of the study:

1. Acute toxicity tests: These are short-term studies, typically lasting a few days to a couple of weeks [33].
2. Subchronic toxicity tests: These studies usually span around 90 days [33,34].
3. Chronic toxicity tests: These long-term studies can last several months to a year or more [33]

### Using AI to Determine the Drug Toxicity of Collagen

AI algorithms can be used to predict toxicity based on the chemical and biological properties of the compounds. AI uses neural networks to analyze molecular graphs or sequences to detect toxicity-related patterns.

The evaluation of pharmacokinetics and toxicity is crucial for designing new therapeutic candidates with in silico virtual screens, and generative AI outputs a vast number of molecules that must be filtered into a tractable number for synthesis and experimental validation. For this case example, the absorption, distribution, metabolism, excretion, and toxicity (ADMET) AI program was used to determine the toxicity of collagen. ADMET is an effective primary filter that evaluates candidate compounds based on their ADMET properties. ADMET-AI is a simple, fast, and accurate digital computer web interface for predicting the ADMET properties of molecules using machine learning models.

**Figure 3.**
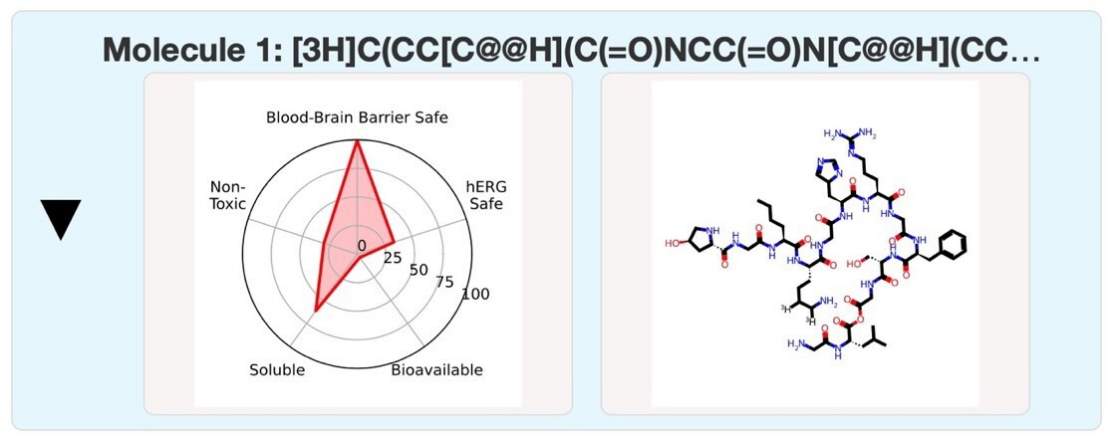
Virtual calculation of the blood-brain barrier. hERG: human Ether-à-go-go–related gene.

The virtual calculation of the blood-brain barrier is shown in Figure 3 [30], which effectively protects the brain tissue from circulating pathogens and other potentially toxic substances. This calculation shows the toxicity of collagen to be low. Collagen itself was shown to be safe and nontoxic.

## Challenges With the Uses of AI for Drug Discovery

Despite promising advancements, several challenges remain for the integration of AI and quantum computing in drug discovery. The ethical implications of using AI in drug discovery must be addressed. Ensuring transparency in AI algorithms and maintaining accountability in decision-making processes are critical to gaining public trust and regulatory approval, which can be achieved by using an explainable AI approach. Furthermore, the potential for bias in AI models necessitates ongoing scrutiny to ensure equitable access to new therapies.

### Data Privacy and Ethics

The use of AI and AI algorithms comes with concern for the privacy and security of user data. Data poising and alterations underlying models put AI users at risk. Implementing federated learning allows for the training of AI models on decentralized data sources without sharing sensitive data. The fully homomorphic encryption technique is used in most federated searching techniques. This is crucial in drug discovery, where patient data and proprietary research information must remain confidential. Federated learning enables collaborative learning across different research institutions or pharmaceutical companies, allowing them to leverage each other’s data without compromising privacy. Since raw data are not centralized, the risk of data breaches is minimized, making it a secure choice for handling sensitive information in drug discovery. Fedrated learning can be integrated with the AI and quantum computing techniques discussed in this paper, enhancing the predictive capabilities while maintaining data integrity and privacy.

### Validation and Accuracy

Computational models must be rigorously validated against experimental data to ensure that their predictions are reliable. This includes demonstrating that computational methods are accurate and can reliably substitute for laboratory-generated data. Multiple stakeholders (eg, academia, industry, and regulatory bodies) would need to validate and reproduce computational data for different types of products.

AI models rely on large, high-quality datasets, whereas pharmaceutical data are often limited, biased, or proprietary, affecting the model’s performance. In addition, AI-generated predictions can lack transparency, making it difficult to understand how a model arrived at a particular conclusion, which is critical in drug development. Although AI predictions can be highly accurate, inconsistencies may still lead to failures in identifying effective drugs or result in overlooking promising candidates. To ensure reliability, AI-driven drug discovery must meet stringent FDA regulatory standards and address ethical concerns, including potential bias in drug development and risks to patient safety. AI models often struggle with the complexity of biological systems, such as multitarget interactions, immune response, and genetic variations. Despite these challenges, AI will continue to improve and is expected to play a significant role in the future of drug discovery. The findings of the present case study were intended to demonstrate that the computational assessment of drug toxicity closely aligns with actual laboratory data. This approach not only replicates laboratory results but does so at a significantly reduced cost.

## SStrengths of the Proposed Approach

Computational models can lower the costs of bringing new drugs to market by reducing the need for extensive animal studies or large human trials. Quantum computing, AI, and machine learning have improved with respect to accuracy and generalizability, and there is growing potential for their application in areas traditionally requiring laboratory data (eg, toxicology and pharmacodynamics). Advances in quantum computing, molecular dynamics, and systems biology would help computational models closely mimic biological systems and make predictions more reliable.

AI and quantum computing facilitate the drug discovery process from the following aspects:

1. Data analysis and pattern recognition: AI algorithms can analyze vast datasets, including genetic Protonix and clinical data to identify potential therapeutic targets and predict drug interactions. This capability allows researchers to uncover disease-associated targets and molecular pathways more efficiently than traditional methods, which often rely on trial and error [35-37].
2. Molecular simulation: Quantum computing enables more accurate simulations of molecular interactions than classical computers [38]. This allows researchers to explore a broader range of potential drug candidates and significantly predict their efficacy and safety, speeding up the drug discovery process [37].
3. Integration of computational models: The combination of AI and quantum computing allows for the development of sophisticated computational models to simulate complex biological systems. This integration can lead to better-informed decisions in drug development and regulatory processes, ultimately enhancing patient safety [35].
4. Reduction of laboratory testing: By using computational data, the need for extensive laboratory and animal testing can be decreased. This not only reduces cost but also shortens the time required to bring new drugs to market [37].
5. Quantifying development costs: The costs are quantified by evaluating the total expenses incurred during the drug development process, including research and development, clinical trials, and regulatory approvals. Traditional methods can take up to 15 years and cost around US $1 billion, whereas quantum computing can potentially reduce this timeline and cost significantly [37].

Researchers may also review case studies where quantum computing has been implemented in drug discovery to assess the financial and temporal savings achieved compared to conventional methods [37].

The burgeoning field of computational data, propelled by AI and quantum computing advancements, stands to revolutionize new drug discovery and approval processes. Computational methods can significantly accelerate the identification of potential drug candidates, predict their efficacy, and assess safety, thereby reducing the traditional time and cost burdens associated with pharmaceutical development. By integrating AI and quantum computing with extensive chemical databases, researchers can efficiently simulate biological interactions, streamline virtual screening, and predict drug toxicity—ultimately enhancing the likelihood of successful drug development. Furthermore, the implications for the FDA regulatory framework are examined, highlighting how computational data can inform and expedite the approval process, leading to faster review cycles and improved postmarket surveillance. This situation calls for a paradigm shift from traditional laboratory methods to data-driven approaches, emphasizing the need for rigorous validation and collaboration among stakeholders to establish robust regulatory standards for computational models in drug discovery.

AI is far cheaper per compound than laboratory-based testing, especially for initial screenings. For example, screening 1000 compounds via AI might cost US $10,000–US $50,000, depending on the computational setup [20]. The same screening using in vitro methods could cost US $1–US $10 million or US $50–US $500 million using in vivo methods once augmented reality AI models are deemed significant. Once developed and validated, these models significantly reduce long-term expenses, making them more cost-effective than laboratory methods for large-scale or preliminary screenings.

As demonstrated with our case study, AI is often used as a first-pass filter to predict drug toxicity, reducing the number of compounds that need to be tested in the laboratory. By prioritizing only those promising candidates for laboratory testing, researchers can combine the speed and cost-effectiveness of AI with the rigor and accuracy of laboratory results, achieving a balance of cost and reliability.

## Summary and Future Prospects

AI and quantum computing offer unique capabilities to tackle complex problems in drug discovery, which is a critical challenge in pharmaceutical research. Regulatory agents will need to adapt to these new technologies. Regulatory processes may become more streamlined, using adaptive clinical trials, accelerating pathways, and better integrating digital data to reduce the time and cost of bringing new drugs to market. Computational data methods could reduce the cost and time involved in experimental drug discovery, allowing researchers to simulate biological interactions and screen large compound libraries more efficiently. Creating virtual data for drug discovery involves several stages, each using specific methods such as simulations, synthetic data generation, data augmentation, and tools to generate, collect, and affect human interaction to identify and develop new drugs. Here, we have emphasized that knowing the molecular structure of a drug is a critical factor in determining its toxicity and for other aspects of the drug discovery and approval process. Using computational data in drug discovery has transformed the pharmaceutical and biotechnology industries by accelerating research, reducing costs and timeliness, and improving the likelihood of success. Overall, the integration of AI and quantum computing represents a transformative shift in drug discovery, offering the potential for faster, more efficient, and more effective therapeutic development. As these technologies continue to evolve, they will likely play a pivotal role in shaping the future of pharmaceuticals. Nevertheless, several research questions remain to be explored to realize this shift, including:

(1) Can AI reliably predict drug toxicity compared to traditional laboratory results? Hypothesis: The incorporation of quantum computing into molecular modeling improves the predictive capabilities of AI, leading to more accurate toxicity assessments.

(2) Does the integration of quantum computing enhance the accuracy of molecular modeling and drug discovery? Hypothesis: The incorporation of quantum computing into molecular modeling improves the predictive capabilities of AI, leading to more accurate toxicity assessments.

(3) How do AI-driven toxicity predictions compare to laboratory outcomes in terms of cost and time efficiency? Hypothesis: Using AI and quantum computing for toxicity prediction significantly reduces the need for laboratory experiments, thereby decreasing both costs and development time in the drug discovery process.

The convergence of AI and quantum computing holds great potential for revolutionizing drug discovery and approval processes. Continued research is needed to refine quantum algorithms and integrate them with AI systems effectively.

## Abbreviations

ADMET: absorption, distribution, metabolism, excretion, and toxicity
AI: artificial intelligence
DHT: digital health technology
FDA: US Food and Drug Administration
HOMO-LUMO: Highest Occupied Molecular Orbital-Lowest Unoccupied Molecular Orbital
IDLH: Immediately Dangerous to Life or Health
ISO: International Organization for Standardization
LD50: median lethal dose
PMMA: polymethyl methacrylate
SMILES: simplified molecular input line entry system
VQE: Variational Quantum Eigensolver
